# Using Positive End‐Expiratory Pressure to Predict and Quantify Tracheobronchomalacia in Grade 3 Bronchopulmonary Dysplasia

**DOI:** 10.1002/ppul.71384

**Published:** 2025-11-12

**Authors:** Stephanie A. Adaikalam, Rebecca S. Rose, Ibrahim A. Sammour, Sarah E. Bauer, Gregory S. Montgomery, A. Ioana Cristea

**Affiliations:** ^1^ Department of Pediatrics Indiana University School of Medicine Indianapolis IN USA

**Keywords:** bronchopulmonary dysplasia, bronchoscopy, oxygen saturation index, positive end‐expiratory pressure, sedation, tracheobronchomalacia

## Abstract

**Objectives:** Tracheobronchomalacia (TBM) is common in Grade 3 Bronchopulmonary Dysplasia (BPD), but there is wide variation in frequency and method of evaluation. Objective quantitative methods are needed for translational studies and future drug trials. This study aimed to determine whether positive end‐expiratory pressure (PEEP) and oxygen saturation index (OSI) can predict TBM in Grade 3 BPD.

**Methods:** We retrospectively reviewed flexible bronchoscopy videos from a cohort of infants with Grade 3 BPD to determine the presence of TBM. PEEP and mean OSI in the week before the first bronchoscopy were assessed as predictors of TBM using multiple logistic regression.

**Subject Demographics:** The study included 56 infants with Grade 3 BPD with a median gestational age (GA) of 25.3 weeks (IQR 2.8) and median post‐menstrual age (PMA) at first bronchoscopy of 40.7 weeks (IQR 6.1).

**Results:** Thirty‐seven subjects (66%) were found to have TBM, although 13 subjects lacked evidence of TBM on the first evaluation (false negative rate 35%). PEEP and OSI had 86% sensitivity for predicting TBM, but only 30% specificity (*p* = 0.02). In a post‐hoc analysis of the 13 initial bronchoscopies that “missed” TBM, 10 had a higher level of sedation in the first evaluation compared to the later assessment that revealed TBM.

**Conclusions:** We found that PEEP and OSI are sensitive but not specific for predicting TBM in Grade 3 BPD. In addition, there are frequent false negative evaluations when bronchoscopy is performed under higher levels of sedation. We propose a more precise bronchoscopy method for quantifying TBM.

## 1 Introduction

Bronchopulmonary dysplasia (BPD) affects over 10,000 infants in the United States per year [1]. Its severity is clinically defined by respiratory support requirements at 36 weeks postmenstrual age (PMA) [2], but does not account for the various contributions of lung parenchymal, airway, or vascular abnormalities in individual patients [3]. Tracheobronchomalacia (TBM), or floppy central airways, affects 30–50% of infants with BPD [4, 5, 6]. This malacia results in obstruction as the airway collapses secondary to the increased intrathoracic pressure during exhalation. There are no efficacious treatments for severe TBM, and the combination of TBM and BPD results in increased risk of prolonged hospitalization, tracheostomy dependence, and death [5, 6, 7, 8].

The true prevalence of TBM is poorly understood because of the wide variation in practices of assessment and quantification. Bronchoscopy is considered the gold standard for diagnosis, but there is a lack of agreement in the literature about factors that influence assessment of TBM, such as type of bronchoscopy (rigid *vs.* flexible), setting of assessment (operating room *vs.* bedside), respiratory support at the time of procedure, and level of patient sedation. In addition, a recent survey of centers participating in the BPD Collaborative showed that only 33% of surveyed centers perform bronchoscopy on “most” infants with severe BPD [9]. Even among centers that routinely perform bronchoscopy, quantifying TBM remains subjective. Most bronchoscopists estimate the severity of TBM by describing a percentage of airway diameter collapse [10]. While inter‐rater reliability for the *presence* of TBM is fair to moderate, inter‐rater reliability for the *severity* of TBM is generally poor, even when assessed at a single center with pre‐determined criteria [11, 12, 13]. Given the subjectivity of quantification by bronchoscopy, some institutions have opted to use computed tomography (CT) scans or magnetic resonance imaging (MRI) to more objectively quantify TBM [14, 15, 16]. Major benefits of these evaluations are that they can usually be performed without sedation, and airway collapse can be more objectively quantified. However, CT scans carry a significant radiation burden in this vulnerable population, and most centers are not equipped with the expertise and technology required for routine clinical MRI airway assessments [17].

These challenges pose significant barriers as we seek new treatment strategies for TBM. Translational work and future drug studies require accurate diagnosis and quantification of TBM. Biomarkers cannot be associated with TBM if the infant′s airway has never been evaluated. The effects of new drugs cannot be accurately described if TBM cannot be quantified at different time points, and data cannot be combined from multi‐center trials without standardized assessments. While there is significant practice variation in ventilator strategies to minimize the effects of TBM, many centers use positive end‐expiratory pressure (PEEP) to combat the increase in intrathoracic pressure during exhalation that causes large airway collapse in the setting of TBM [18]. However, PEEP alone cannot be used to predict or quantify TBM, as infants with severe parenchymal disease (in the absence of TBM) may also require high PEEP to support oxygenation through increased alveolar recruitment. The combination of PEEP with a measure of hypoxemia burden has potential as a tool to predict TBM. Oxygenation index (OI) (OI = mean airway pressure [MAP] x Fraction of Inspired Oxygen [FiO_2_] x 100 ÷ Partial Pressure of Arterial Oxygen [PaO_2_]) is routinely used in intensive care units to quantify the severity of hypoxemic respiratory failure and guide decisions for necessary interventions, such as initiation of inhaled nitric oxide when OI is greater than 25 [19] or initiation of extracorporeal membrane oxygenation when OI is greater than 40 [20]. The use of OI is limited by the necessity of an arterial line for frequent sampling of PaO_2_. Alternatively, oxygen saturation index (OSI) (OSI = MAP x FiO_2_ x 100 ÷ oxygen saturation (SpO_2_)) can be calculated based on routine vitals and ventilator settings without invasive arterial samples. Muniraman et al. demonstrated that OSI correlates well with oxygenation index (OI) in neonates and proposed an equation for deriving OI from OSI (OI = 2x OSI) [21]. Here, we hypothesize that average PEEP and OSI in the week before any evaluation of TBM can be used as predictors of TBM. In addition, we propose an improved, more precise bronchoscopic method for quantifying TBM in the setting of Grade 3 BPD.

## 2 Materials and Methods

### 2.1 Subject Screening and Clinical Details

A RedCap database [22] of infants admitted to the neonatal intensive care unit (NICU) at Riley Hospital for Children was screened for infants with Grade 3 BPD born between 2022 and 2024 who received flexible bronchoscopy during their NICU hospital course. Rigid bronchoscopies were not included. Clinical details, including gestational age (GA) at birth, birth weight, and PMA at the time of bronchoscopy, were collected. In addition, the PEEP and mean OSI in the week before the first bronchoscopy were recorded. This study was approved by the Institutional Review Board of Indiana University (IRB# 1710518896). A waiver of informed consent was granted due to the retrospective nature of the study with minimal risks.

### 2.2 Bronchoscopy Review

Bronchoscopy videos stored in VaultStream (Olympus) were edited to remove any patient identifiers, and two blinded pulmonologists independently reviewed each video. The blinded reviewers were instructed to rate each of 3 segments (distal trachea, right mainstem bronchus, and left mainstem bronchus) as either < 50% collapse or > 50% collapse, regardless of the shape or location of the collapse (e.g., posterior membrane intrusion vs anterior cartilage collapse). If there was discordance between the two reviewers, a third blinded reviewer evaluated the video. Each subject was labeled as having TBM if greater than 50% collapse was present in any of the three segments. If TBM was not present on a subject′s first bronchoscopy, and the subject had received additional flexible bronchoscopies during the same admission, these subsequent assessments were similarly evaluated. If any bronchoscopy identified TBM, then no further bronchoscopies were analyzed. The subject was identified as having TBM if more than 50% collapse was present on any flexible bronchoscopy during the initial hospital admission. In addition to the blind review of the bronchoscopy videos, details from each assessment, including bronchoscope size, location (OR *vs.* bedside), procedure‐related sedation used, and ventilator settings during the procedure, were collected.

## 3 Statistical Analysis

Demographic and clinical details were reported as a percentage for categorical data or median with interquartile range for quantitative data. The Mann‐Whitney U test was used to compare the “TBM” and “no TBM” groups for demographic differences because of non‐normal distributions. Chi‐square test was used for categorical data. To assess inter‐rater reliability of the two blinded bronchoscopy reviews, Cohen's Kappa was calculated. Multiple logistic regression was used to predict the presence of TBM using PEEP and mean OSI in the week before the first bronchoscopy. OSI was divided into 3 categories: low OSI (< 8), moderate OSI (8–12), and high OSI (> 12). The prediction model used 50% probability as the cutoff for assigning TBM. Model characteristics were also calculated.

## 4 Results

Clinical data were collected from 56 infants with Grade 3 BPD who received flexible bronchoscopy. This cohort had a median GA of 25.3 weeks (IQR 2.8), and the median PMA at time of first bronchoscopy was 40.7 weeks (IQR 6.1). Table 1 includes further Demographic details. The median PEEP before evaluation was 12 cmH_2_O (IQR 4), ranging 6–18 cmH_2_O.

**Table 1:** Subject demographics (*N* = 56).

Clinical values	No TBM (*n* = 19, 34%)	TBM (*n* = 37, 66%)	*p* value
Male sex	*n* = 7 (37%)	*n* = 20 (54%)	0.22
	**Median (IQR)**	**Median (IQR)**	
GA at birth (weeks)	25 (2.5)	25.9 (2.8)	0.32
Birth weight (grams)	720 (317)	685 (280)	0.32
PEEP before first bronchoscopy (cmH_2_O)	10.5 (4.3)	13 (4.25)	0.07
OSI before first bronchoscopy	5.6 (4.1)	6.7 (2.9)	0.57
PMA at first bronchoscopy (weeks)	40 (5.7)	41 (5.9)	0.12

Abbreviations: GA = gestational age, OSI = oxygen saturation index, PEEP = positive end‐expiratory pressure, PMA = postmenstrual age, TBM = tracheobronchomalacia.

Blind review of each infant'′s first bronchoscopy resulted in 23 infants with TBM (41%), as defined by more than 50% collapse. However, when including subsequent bronchoscopies that revealed TBM, 37 (66%) subjects were found to have TBM. Therefore, the first bronchoscopy “missed” TBM 35% of the time (false negative rate). When considering inter‐rater reliability for assessment of > 50% collapse in any large airway, there was 79% agreement between the initial two reviewers (κ = 0.57, moderate agreement) [12].

The multiple logistic regression produced a model for predicting TBM with 86% sensitivity and 30% specificity (*p* = 0.02). The area under the receiver operator curve (AUC‐ROC) was 0.69. For each single‐digit increase in PEEP, an infant'′s probability of having TBM increased by 36% (odds ratio of 1.36). Having a moderate (8–12) or high (> 12) OSI decreased the probability of TBM by 55% or 76% (odds ratios of 0.45 and 0.24), respectively.

**Figure 1:**
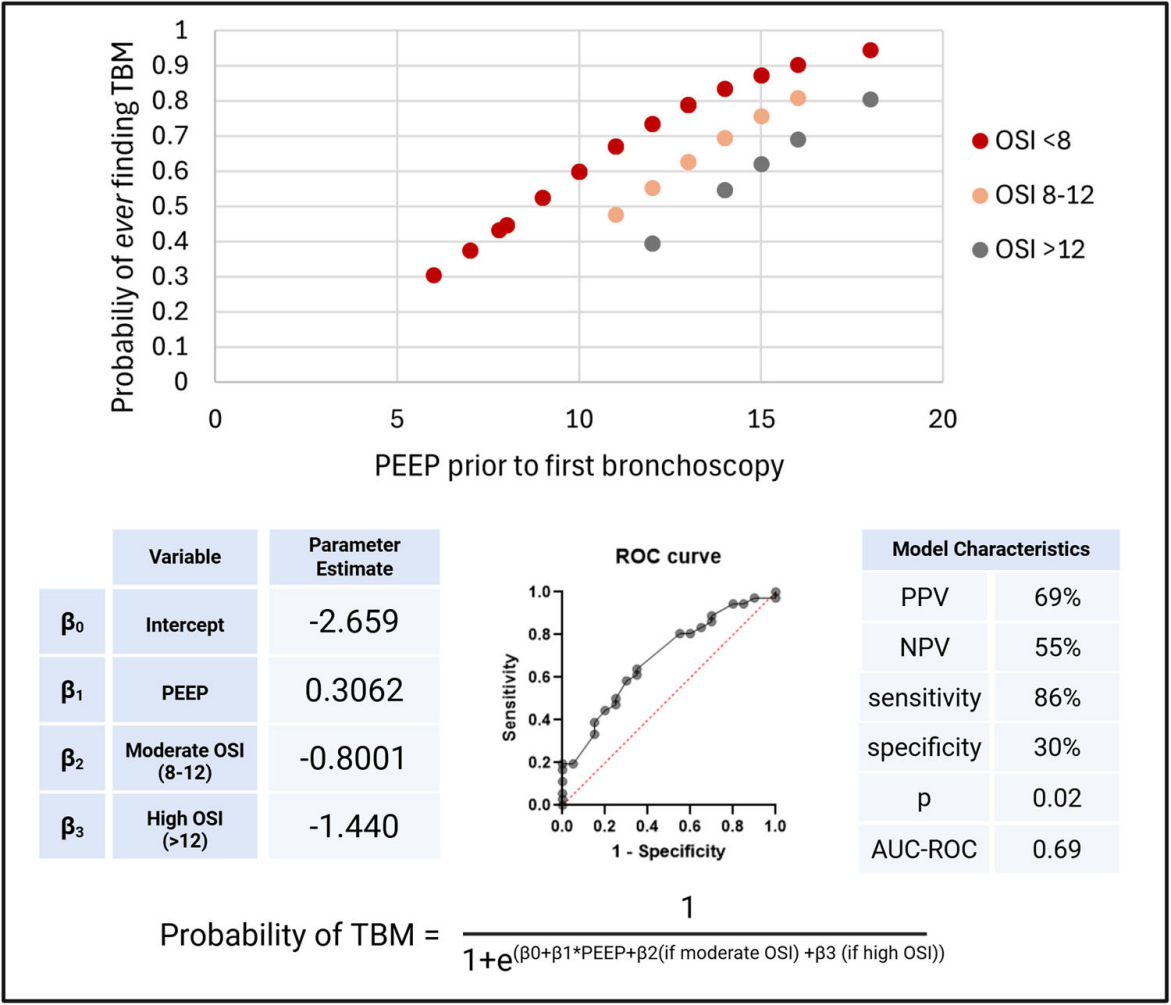
PEEP and OSI as Predictors of TBM: (Top) Prediction model showing probability of ever finding TBM (y‐axis) based on PEEP before first bronchoscopy (x‐axis), separated by mean OSI in the week before the first bronchoscopy. Subjects with low OSI and high PEEP have higher probability of having TBM. The receiver operator curve is shown below with parameter estimates and model characteristics. Created in BioRender. Adaikalam, S. (2025) https://BioRender.com/xiddslz. [Color figure can be viewed at wileyonlinelibrary.com]

Figure 1 demonstrates the prediction model and its characteristics.

**Figure 2:**
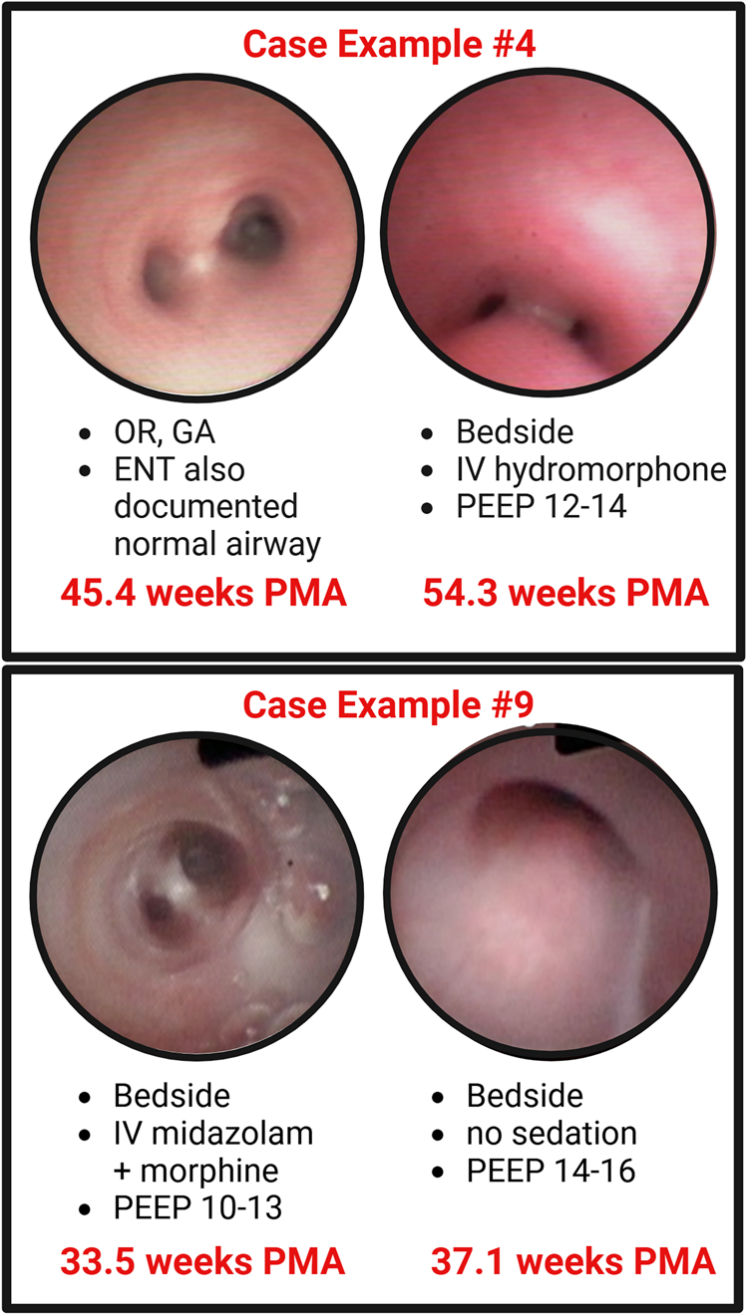
Case examples of TBM “missed” on first bronchoscopy: Each black box provides a pictorial example of a case with no evidence of TBM on first assessment (left) and severely obstructing TBM found on a subsequent evaluation (right). Created in BioRender. Adaikalam, S. (2025) https://BioRender.com/djvinw8. [Color figure can be viewed at wileyonlinelibrary.com]

After finding a high false negative rate on the first bronchoscopy assessment, we further compared differences in the bronchoscopy setting when evidence of TBM was absent compared to subsequent assessments where it was present. We found that in 10 of 13 cases (77%), there was a higher level of sedation during the first procedure compared to subsequent evaluation. Seven of the “missed” cases (54%) took place in the operating room under general anesthesia, where TBM was later identified during a bedside evaluation while using IV sedatives such as opioids or benzodiazepines (4 cases) or with minimal sedation (either enteral medications or no sedation, 3 cases). Another 3 cases of TBM (23%) were “missed” during bedside evaluations using IV sedatives, but later identified TBM when assessed with enteral or no sedation. The median time between the bronchoscopy with no evidence of TBM and the evaluation that identified TBM was 6.7 weeks (IQR 6.4). Table 2 provides details for each case of “missed” TBM, where each line shows the PMA, location, and sedation medications for each bronchoscopy. In addition, Figure 2 demonstrates two pictorial examples, with the first evaluation under increased sedation with no evidence of TBM (left) compared to a later evaluation with minimal sedation showing severely obstructing TBM (right).

**Table 2:** Case examples of TBM “Missed” on first evaluation.

Case examples	PMA (weeks)	Location	Sedation	Findings
Evidence of TBM Absent During OR Evaluation (GA)				
1a	43.4	OR	general anesthesia	No evidence of TBM
1b	45.9	NICU	IV benzodiazepine + morphine	TM
2a	41.0	OR	general anesthesia	No evidence of TBM
2b	60.6	PICU	IV ketamine	TM
3a	33.4	OR	general anesthesia	No evidence of TBM
3b	39.7	NICU	no sedation	TM
4a	45.4	OR	general anesthesia	No evidence of TBM
4b	54.3	PICU	IV hydromorphone	TM
5a	39.8	OR	general anesthesia	No evidence of TBM
5b	51.8	PICU	IV hydromorphone + benzodiazepine	No evidence of TBM
5c	55.0	PICU	po benzodiazepine	TBM
6a	46.1	OR	general anesthesia	No evidence of TBM
6b	51.9	PICU	IV dexmedetomidine + morphine	TM
7a	44.7	OR	general anesthesia	No evidence of TBM
7b	53.7	PICU	po benzodiazepine	TM
Evidence of TBM Absent During Bedside Evaluation (IV sedation)				
8a	40.6	NICU	IV dexmedetomidine + morphine	No evidence of TBM
8b	47.3	NICU	no sedation	TM
9a	33.5	NICU	IV benzodiazepine + morphine	normal
9b	37.1	NICU	no sedation	TM
10a	49.4	PICU	IV benzodiazepine + morphine	No evidence of TBM
10b	51.0	PICU	IV dexmedetomidine	No evidence of TBM
10c	53.0	PICU	po benzodiazepine	TBM
Evidence of TBM Absent During First Evaluation (Other)				
11a	40.0	NICU	IV dexmedetomidine + benzodiazepine	No evidence of TBM
11b	61.0	PICU	IV ketamine	TM
12a	33.8	NICU	po morphine	No evidence of TBM
12b	43.8	NICU	no sedation	TM
13a	30.5	NICU	IV morphine	No evidence of TBM
13b	41.4	OR	general anesthesia	TM

*Note:* Each case (*n* = 13) is an example of TBM “missed” on first bronchoscopy assessment. Each row represents a separate evaluation, demonstrating differences in location, sedation provided, and findings of the procedure. The first bronchoscopy of cases 1−7 took place in the operating room (OR) under general anesthesia (GA). The first evaluation of cases 8−10 took place at the bedside under IV sedation, with subsequent evaluation under minimal sedation. Cases 11−13 did not have a clear difference in sedation medications present at the time of the first and second evaluation.

## 5 Discussion

Infants with severe BPD and concomitant TBM have increased morbidity and mortality. With no good TBM treatment options, families of children requiring ongoing high‐level respiratory support are left to decide between invasive mechanical ventilation at home via tracheostomy or redirection of care. Basic and translational research is needed to further our understanding of the underlying mechanisms of TBM and provide new TBM‐specific treatment strategies. Therefore, it is critical to be able to predict which infants have TBM and accurately quantify it when it is identified.

Here we have described PEEP and OSI as sensitive predictors of TBM in the setting of BPD, where high PEEP in the setting of low OSI increases the probability of the presence of TBM. With 86% sensitivity, PEEP and OSI can be used as a tool to guide clinicians to further evaluation. In addition, the prediction model also allows researchers to correlate biomarkers and other findings with an infant'′s probability of having TBM, even in the absence of bronchoscopy or imaging data.

In addition to using PEEP and OSI as predictors of TBM, future research needs more precise quantification of TBM severity. Several studies have shown poor inter‐rater reliability when estimating severity by percentage of airway diameter collapse [11, 12]. In addition, the degree of collapse is dependent upon the level of sedation, PEEP provided during the procedure, and other factors such as bronchoscope size and type (rigid *vs.* flexible scope). While anesthesia literature shows worsening of pharyngeal obstruction with deeper sedation [23, 24], to our knowledge this is the first work to describe the impact of sedation on assessment of TBM. We report here retrospective examples of TBM assessment with worse large airway collapse in the setting of *less* sedation. While pharyngeal obstruction is secondary to decreased tone, which is further decreased in the setting of anesthesia (or sleep), the obstruction resulting from TBM is caused by the increased intrathoracic pressure with exhalation. This intrathoracic pressure is reduced when an infant is sedated, but intrathoracic pressure is increased during baseline activities such as crying or having a bowel movement. Our data show a high rate (35%) of false negative assessments during the first bronchoscopy, which were often performed under higher levels of sedation. This data, in addition to the physiology of the obstruction resulting from TBM, shows that TBM assessments are best performed under the minimum sedation (if any) that is safe and clinically tolerable for a patient.

**Figure 3:**
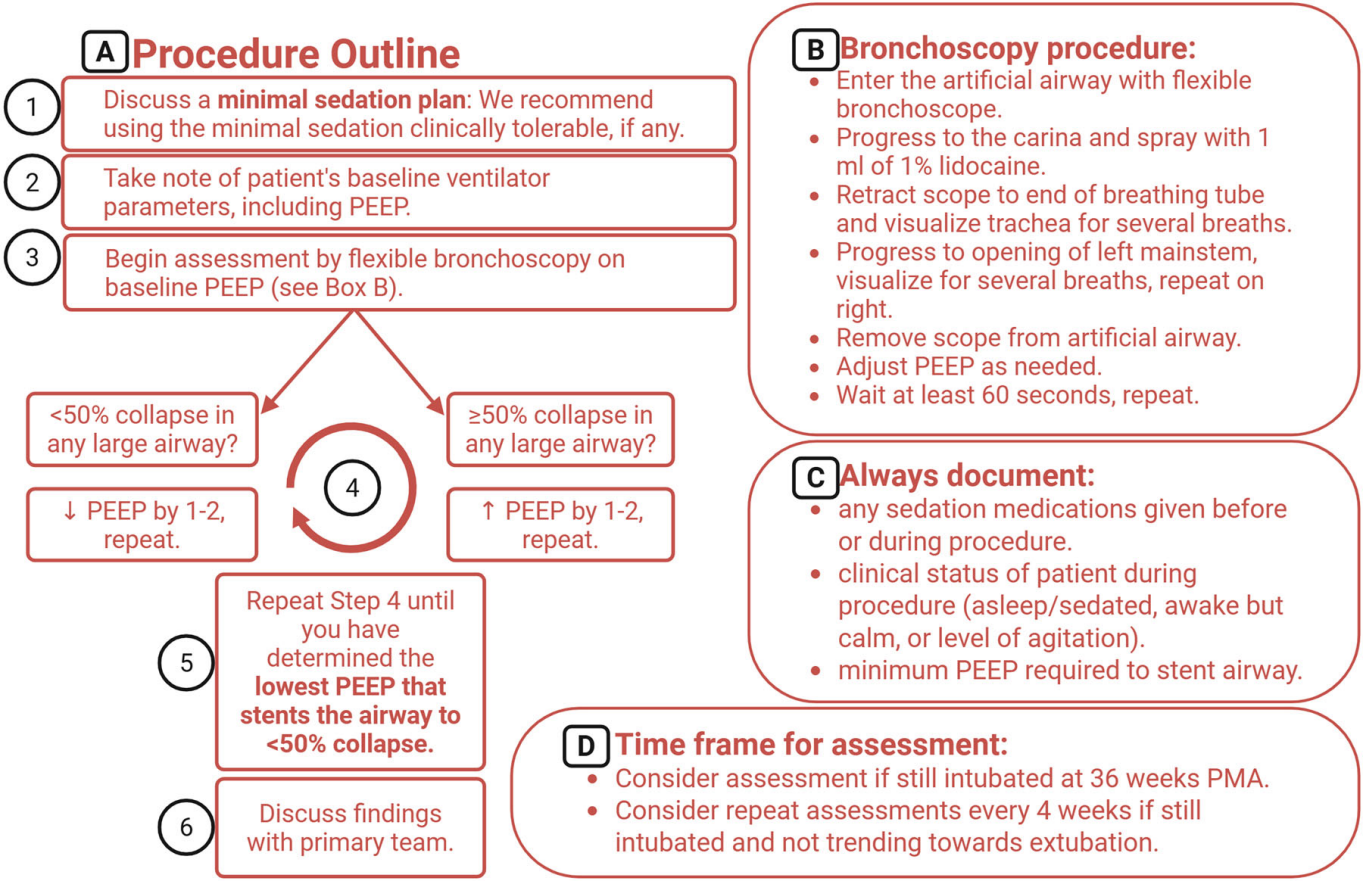
Quantifying tracheobronchomalacia in grade 3 BPD using minimum PEEP required to stent the airways: The flow chart shows a new proposed method for quantifying TBM using repeated assessments while varying the PEEP to find the “**minimum PEEP required to stent the airway”** to no more than 50% collapse. We recommend completing the evaluation using the minimum sedation clinically tolerable for the individual patient. Created in BioRender. Adaikalam, S. (2025) https://BioRender.com/doi6j2p. [Color figure can be viewed at wileyonlinelibrary.com]

In addition to minimizing sedation, bronchoscopists must consider the ventilator support a subject is receiving at the time of evaluation [25]. We cannot compare 90% collapse in one infant on a PEEP of 8 with 50% collapse in another infant on a PEEP of 15. They likely both have severely obstructing TBM. In addition, we know there is poor inter‐rater reliability when estimating a specific percentage of airway collapse, but better agreement when simply deciding the binary presence of > 50% collapse or not. Here we propose an improved, more precise method for assessing TBM in the setting of Grade 3 BPD (Figure 3). Rather than estimating the percentage of airway diameter collapse on an arbitrary level of ventilatory support and sedation, we propose serial evaluation with varying levels of PEEP until the bronchoscopist has determined the **“minimum PEEP required to stent the airways”** to no more than 50% collapse, while under the minimum sedation that is safe and tolerable for that individual patient. If there is less than 50% collapse on a physiologic PEEP (i.e., 5–8), we consider that infant to have no tracheobronchomalacia (assuming minimal sedation was used and the infant was sufficiently arousable during the assessment). For infants with more than 50% collapse, the “minimum PEEP required to stent the airways” serves as the quantifying measure, enabling numerical quantification across the spectrum of disease severity. This quantitative metric facilitates comparison between different subjects, while also allowing changes to be tracked within the same subject over time (e.g. to measure response in a future drug trial).

Some centers preferentially consider rigid bronchoscopy for evaluation of TBM. We intentionally excluded rigid bronchoscopy from the current work, and we do not recommend rigid bronchoscopy as the primary assessment for TBM in the setting of BPD. Hysinger et al. have previously shown 75% agreement between rigid and flexible scopes when evaluating for the presence of TBM, with poor correlation for tracheomalacia severity [13]. However, both evaluations were performed under the same general anesthesia. It is known that the introduction of a flexible bronchoscope does increase auto‐PEEP [26], but this effect is likely more significant in the setting of rigid bronchoscopy, as the larger diameter rigid scope stents open the airway and provides greater resistance to passive exhalation. In addition, rigid bronchoscopy requires general anesthesia, which our data have demonstrated to negatively impact the assessment of TBM. For these reasons, we highly recommend flexible bronchoscopy (under minimal, if any, sedation) for a complete dynamic assessment of TBM.

While this is the first available prediction model for TBM, it has several limitations. Firstly, the model uses PEEP, which has been clinically titrated before any airway evaluation (which could include titration of PEEP under visualization of the airway). There is wide variation and debate about the levels of PEEP needed in severe BPD. Our multi‐disciplinary BPD team uses PEEP to combat the intrathoracic pressure produced with exhalation that causes large airway collapse in the setting of suspected TBM [18, 25, 27], allowing for complete exhalation and improved patient‐ventilator interactions [18, 28]. In contrast, some centers use a “low PEEP” strategy. For example, in recent work, Yat‐Ting Su et al. report increased PIP as a significant predictor of TBM, but PEEP was not significantly related to a TBM diagnosis. Importantly, authors report a mean PEEP of 6.7 cmH_2_O in TBM vs 4.1 cmH_2_O in no TBM. [5] This is an important contrast to our cohort, whose median PEEP before first bronchoscopy in subjects with TBM was 13 cmH_2_O versus 10.5 cmH_2_O in those without TBM (*p* = 0.07). While the increase in the TBM group is not statistically significant, the range of PEEP demonstrates the differences in PEEP strategies at different institutions. The proposed prediction model will be most applicable at centers that utilize a high‐PEEP strategy similar to our institution. In addition, this model is based on retrospective review of bronchoscopies at one center, which took place under variable settings (OR *vs.* bedside), variable levels of sedation (or without sedation), various ventilator settings, etc. Future prospective, multi‐center work is needed to validate these findings. Imaging assessments (i.e., CT or MRI) of TBM were not available for the described cohort, but we intend to include measurements of TBM via ultrashort echo‐time (UTE) magnetic resonance imaging (MRI) [16] as a validation tool for prospective studies of the proposed bronchoscopy quantification protocol.

We have provided case examples of TBM identified on subsequent bronchoscopies, when the initial bronchoscopy performed under a higher level of sedation showed no evidence of TBM. Of the 13 cases that were diagnosed after the first evaluation, 77% of these cases had a higher level of sedation in the first evaluation. We cannot be certain that TBM was “missed”, as it is plausible that TBM developed in the time frame between evaluations. Similarly, in this retrospective study, subsequent bronchoscopies were not reviewed if a prior evaluation diagnosed TBM. Therefore, we cannot comment on changes in TBM over time. While there is limited literature demonstrating the time frame for development and progression of tracheobronchomalacia in BPD, Mohanakrishnan et al. recently showed no difference in serial MRI measurements of tracheomalacia, when first evaluated near 41 weeks PMA with subsequent evaluation 4−8 weeks later [29]. Similarly, our cohort had a median PMA of 40.7 weeks at the time of first bronchoscopy, with a median of 6.7 weeks between evaluations. Based on this MRI data and the general clinical consensus that TBM *improves* with age, it is unlikely that subjects in our cohort with no evidence of TBM developed severely obstructing TBM in this short period. While this points to sedation as a *possible* cause of “false negative” TBM evaluations, prospective work is needed to establish a certain causative link, including understanding the effects of specific medication classes.

The proposed bronchoscopy assessment (Figure 3) provides standard recommendations to consider when evaluating for TBM, along with a novel method for quantifying TBM based on the “**minimum PEEP required to stent the airways**”. These recommendations have been made based on retrospective review and other published literature described above. Further prospective evaluation of this recommended assessment is needed. In addition, while the proposed assessment recommends reducing sedation to the minimum tolerable, results will still be impacted by any sedation the patient does receive. It is critical that bronchoscopists precisely document the level of sedation that is present during an evaluation, and this information should be considered when describing severity. Although further prospective studies are needed to fully understand the impact of different sedation medications on TBM, at this time we recommend at minimum recording the route (IV vs enteral) and drug class (e.g. opioids, benzodiazepines, general anesthetics, or neuromuscular blockade), as well as a clinical assessment of the patient during the procedure (e.g. sleeping throughout, calm but awake, or degree of agitation). This context will be important when comparing across time points. At our institution, we never reduce sedation from a patient′s clinical baseline for the purposes of the procedure, but we avoid giving extra boluses or enteral medications for the purpose of the procedure itself. For example, if an infant requires IV sedation clinically, our bedside bronchoscopy is performed under that same IV sedation, but we must consider this when discussing the minimum PEEP required to stent the airway. We often counsel our NICU colleagues that if, for example, the infant requires a PEEP of 12 to stent the airways while receiving clinical sedation, they may require higher PEEP if sedation is weaned. In addition, we find that if the bronchoscopy demonstrates a need for higher PEEP than what the infant is currently receiving, sometimes increasing the PEEP to meet their respiratory needs actually *decreases* their sedation needs, since the airway is more able to tolerate mild agitation with routine care.

Infants with severe BPD and TBM have minimal treatment options, and moving the field forward requires sensitive prediction of TBM and accurate quantification. Here we propose a prediction model for TBM using PEEP and OSI. In addition, we propose an improved bronchoscopic method for quantifying TBM in the setting of Grade 3 BPD, allowing for comparison across multiple centers and time points.

## Author Contributions

**Stephanie A Adaikalam:** conceptualization, investigation, writing – original draft, methodology, visualization, writing – review and editing, formal analysis, data curation, supervision. **Rebecca S Rose:** conceptualization, writing – review and editing, visualization, project administration, supervision, resources. **Ibrahim A Sammour:** writing – review and editing, conceptualization, visualization. **Sarah E Bauer:** writing – review and editing, conceptualization, methodology, validation, visualization. **Gregory S Montgomery:** writing – review and editing, conceptualization, validation, methodology. **A Ioana Cristea:** writing – review and editing, conceptualization, validation, methodology, visualization, project administration, supervision.

## Conflicts of Interest

The authors declare no conflicts of interest.

## Data Availability

The data that support the findings of this study are available from the corresponding author upon reasonable request.

## References

[1] E. A. Jensen , E. M. Edwards , L. T. Greenberg , R. F. Soll , D. E. Y. Ehret , and J. D. Horbar , “Severity of Bronchopulmonary Dysplasia Among Very Preterm Infants in the United States,” Pediatrics 148, no. 1 (2021): e2020030007, 10.1542/peds.2020-030007. 34078747 PMC8290972

[2] E. A. Jensen , K. Dysart , M. G. Gantz , et al., “The Diagnosis of Bronchopulmonary Dysplasia in Very Preterm Infants. An Evidence‐Based Approach,” American Journal of Respiratory and Critical Care Medicine 200, no. 6 (2019): 751–759, 10.1164/rccm.201812-2348OC. 30995069 PMC6775872

[3] K. Y. Wu , E. A. Jensen , A. M. White , et al., “Characterization of Disease Phenotype in Very Preterm Infants With Severe Bronchopulmonary Dysplasia,” American Journal of Respiratory and Critical Care Medicine 201, no. 11 (2020): 1398–1406, 10.1164/rccm.201907-1342OC. 31995403 PMC7258644

[4] E. Hysinger , N. Friedman , E. Jensen , H. Zhang , and J. Piccione , “Bronchoscopy in Neonates With Severe Bronchopulmonary Dysplasia In the Nicu,” Journal of Perinatology 39, no. 2 (2019): 263–268, 10.1038/s41372-018-0280-y. 30518799

[5] Y. T. Su , C. C. Chiu , S. H. Lai , et al., “Risk Factors for Tracheobronchomalacia in Preterm Infants With Bronchopulmonary Dysplasia,” Frontiers in Pediatrics 9 (2021): 697470, 10.3389/fped.2021.697470. 34249821 PMC8270074

[6] E. B. Hysinger , N. L. Friedman , M. A. Padula , et al., “Tracheobronchomalacia Is Associated With Increased Morbidity in Bronchopulmonary Dysplasia,” Annals of the American Thoracic Society 14, no. 9 (2017): 1428–1435, 10.1513/AnnalsATS.201702-178OC. 28622012 PMC5711403

[7] S. A. Adaikalam , N. S. Higano , E. B. Hysinger , et al., “Tracheostomy Prediction Model in Neonatal Bronchopulmonary Dysplasia via Lung and Airway MRI,” Pediatric Pulmonology 57, no. 4 (2022): 1042–1050, 10.1002/ppul.25826. 35029053 PMC8930535

[8] S. M. Zak , I. S. Onge , N. S. Higano , et al., “Clinical Outcomes Through Two Years for Infants With Bronchopulmonary Dysplasia and Tracheomalacia,” Pediatric Pulmonology 60, no. 1 (2025): e27383, 10.1002/ppul.27383. 39636156 PMC11758772

[9] M. Bansal , W. M. Manimtim , A. Agarwal , et al., “Outcomes of Ventilator‐Dependent Children With Severe Bronchopulmonary Dysplasia and Tracheobronchomalacia,” Pediatric Pulmonology 60, no. 4 (2025): e71100, 10.1002/ppul.71100. 40243389

[10] C. Wallis , E. Alexopoulou , J. L. Antón‐Pacheco , et al., “ERS Statement on Tracheomalacia and Bronchomalacia in Children,” European Respiratory Journal 54, no. 3 (2019): 1900382, 10.1183/13993003.00382-2019. 31320455

[11] F. Zirek , G. Özcan , M. N. Tekin , et al., “Intra‐Observer and Interobserver Consistency in the Diagnosis of Lower Airway Malacia Using Dynamic Flexible Bronchoscopy in Pediatric Patients,” Pediatric Pulmonology 60, no. 4 (2025): e71099, 10.1002/ppul.71099. 40257402

[12] G. Burg , M. M. Hossain , R. Wood , and E. B. Hysinger , “Evaluation of Agreement on Presence and Severity of Tracheobronchomalacia by Dynamic Flexible Bronchoscopy,” Annals of the American Thoracic Society 18, no. 10 (2021): 1749–1752, 10.1513/AnnalsATS.202009-1142RL. 34000226

[13] E. B. Hysinger , C. K. Hart , G. Burg , A. De Alarcon , and D. Benscoter , “Differences in Flexible and Rigid Bronchoscopy for Assessment of Tracheomalacia,” Laryngoscope 131, no. 1 (2021): 201–204, 10.1002/lary.28656. 32282085

[14] C. Montoya , R. Steinhorn , J. Berger , H. Haroyan , M. Said , and G. F. Perez , “Dynamic Peep Study: A Non‐Invasive Diagnostic Exam to Assess for Effective Peep in Children With Severe BPD,” Lung 200, no. 1 (2022): 59–65, 10.1007/s00408-021-00497-9. 35013755

[15] C. P. Pugh , S. Ali , A. Agarwal , D. N. Matlock , and M. Sharma , “Dynamic Computed Tomography for Evaluation of Tracheobronchomalacia in Premature Infants With Bronchopulmonary Dysplasia,” Pediatric Pulmonology 58 (2023): 3255–3263, 10.1002/ppul.26652. 37646125 PMC10993911

[16] E. B. Hysinger , A. J. Bates , N. S. Higano , et al., “Ultrashort echo‐time MRI for the Assessment of Tracheomalacia in Neonates,” Chest 157, no. 3 (2020): 595–602, 10.1016/j.chest.2019.11.034. 31862439 PMC7118245

[17] J. A. Tkach , S. L. Merhar , B. M. Kline‐Fath , et al., “MRI in the Neonatal ICU: Initial Experience Using a Small‐Footprint 1.5‐t System,” American Journal of Roentgenology 202, no. 1 (2014): W95–W105, 10.2214/ajr.13.10613. 24370170

[18] S. Davis , M. Jones , J. Kisling , C. Angelicchio , and R. S. Tepper , “Effect of Continuous Positive Airway Pressure on Forced Expiratory Flows in Infants With Tracheomalacia,” American Journal of Respiratory and Critical Care Medicine 158, no. 1 (1998): 148–152, 10.1164/ajrccm.158.1.9711034. 9655721

[19] S. G. Golombek and J. N. Young , “Efficacy of Inhaled Nitric Oxide for Hypoxic Respiratory Failure in Term and Late Preterm Infants by Baseline Severity of Illness: A Pooled Analysis of Three Clinical Trials,” Clinical Therapeutics 32, no. 5 (2010): 939–948, 10.1016/j.clinthera.2010.04.023. 20685502

[20] K. Fletcher , R. Chapman , and S. Keene , “An Overview of medical ECMO for Neonates,” Seminars in Perinatology 42, no. 2 (2018): 68–79, 10.1053/j.semperi.2017.12.002. 29336834

[21] H. K. Muniraman , A. Y. Song , R. Ramanathan , et al., “Evaluation of Oxygen Saturation Index Compared With Oxygenation Index in Neonates With Hypoxemic Respiratory Failure,” JAMA Network Open 2, no. 3 (2019): e191179, 10.1001/jamanetworkopen.2019.1179. 30924897 PMC6450323

[22] P. A. Harris , R. Taylor , R. Thielke , J. Payne , N. Gonzalez , and J. G. Conde , “Research Electronic Data Capture (Redcap)‐‐A Metadata‐Driven Methodology and Workflow Process for Providing Translational Research Informatics Support,” Journal of Biomedical Informatics 42, no. 2 (2009): 377–381, 10.1016/j.jbi.2008.08.010. 18929686 PMC2700030

[23] R. G. Evans , M. W. Crawford , M. D. Noseworthy , and S. J. Yoo , “Effect of Increasing Depth of Propofol Anesthesia on Upper Airway Configuration in Children,” Anesthesiology 99, no. 3 (2003): 596–602, 10.1097/00000542-200309000-00014. 12960543

[24] P. R. Eastwood , I. Szollosi , P. R. Platt , and D. R. Hillman , “Collapsibility of the Upper Airway During Anesthesia With Isoflurane,” Anesthesiology 97, no. 4 (2002): 786–793, 10.1097/00000542-200210000-00007. 12357141

[25] R. W. Miller , M. M. Pollack , T. M. Murphy , and R. J. Fink , “Effectiveness of Continuous Positive Airway Pressure in the Treatment of Bronchomalacia in Infants: A Bronchoscopic Documentation,” Critical Care Medicine 14, no. 2 (1986): 125–127, 10.1097/00003246-198602000-00009. 3510811

[26] D. Hsia , R. M. DiBlasi , P. Richardson , D. Crotwell , J. Debley , and E. Carter , “The Effects of Flexible Bronchoscopy on Mechanical Ventilation in a Pediatric Lung Model,” Chest 135, no. 1 (2009): 33–40, 10.1378/chest.08-1000. 18812449

[27] R. K. Kanter , M. M. Pollack , W. W. Wright , and K. M. Grundfast , “Treatment of Severe Tracheobronchomalacia With Continuous Positive Airway Pressure (CPAP),” Anesthesiology 57, no. 1 (1982): 54–56, 10.1097/00000542-198207000-00017. 7046516

[28] N. Napolitano , K. Jalal , J. M. McDonough , et al., “Identifying and Treating Intrinsic Peep in Infants With Severe Bronchopulmonary Dysplasia,” Pediatric Pulmonology 54, no. 7 (2019): 1045–1051, 10.1002/ppul.24328. 30950245

[29] M. Mohanakrishnan , S. R. Munidasa , N. S. Higano , et al., “Serial MRI Evaluation of Tracheomalacia Changes in Neonates With Bronchopulmonary Dysplasia,” American Journal of Respiratory and Critical Care Medicine 211(2025): A1284, 10.1164/ajrccm.2025.211.Abstracts.A1284.

